# Short-term inhibition of fibrinolytic system restores locomotor function after spinal cord injury in mice

**DOI:** 10.1038/s41598-019-52621-8

**Published:** 2019-11-05

**Authors:** Yasuyuki Shiraishi, Atsushi Kimura, Osamu Matsuo, Yoichi Sakata, Katsushi Takeshita, Tsukasa Ohmori

**Affiliations:** 1 Department of Orthopaedics, Jichi Medical University School of Medicine, Tochigi, 329-0498 Japan; 2 Kindai University Faculty of Medicine, Osakasayama, 589-8511 Japan; 3 Department of Biochemistry, Jichi Medical University School of Medicine, Tochigi, 329-0498 Japan

**Keywords:** Spinal cord injury, Spinal cord diseases

## Abstract

Spinal cord injury (SCI) is caused by an initial mechanical insult followed by a series of deleterious events that promote the progressive damage of affected tissues. Fibrinolysis, the process by which plasmin degrades cross-linked fibrin clots, has numerous functions in the central nervous system. However, the roles of the fibrinolytic system in SCI pathophysiology remain unknown. We investigated the roles of fibrinolysis in SCI, and explored therapeutic applications targeting fibrinolysis. Plasminogen-deficient (*Plg*^−/−^) mice exhibited significantly improved locomotor function in the early phase of SCI (the first 7 days post injury), with significant inhibition of bleeding and vascular permeability, but failed to demonstrate conclusive functional recovery. Consistent with these findings, the short-term administration of tranexamic acid (TXA) in wild-type mice over the first 3 days post injury significantly improved locomotor function after SCI, whereas prolonged TXA administration did not. Prolonged TXA administration resulted in significantly lower levels of matrix metalloproteinase activities in the spinal cord, suggesting that inhibition of the fibrinolytic system impaired tissue remodeling. Our results indicate that the fibrinolytic system has time-dependent biphasic actions following SCI. The temporally optimised modulation of fibrinolytic activity may thus be a novel therapeutic strategy to improve functional outcomes after SCI.

## Introduction

Spinal cord injury (SCI) is a devastating condition that often affects young and active individuals. SCI typically leads to persistent dysfunctions below the level of injury, including motor deficits, neuropathic pain, and neurogenic urinary tract dysfunction^1,2^. Experimental models and clinical observations of SCI support the concept of two-phased injury mechanisms, in which the initial mechanical insult (primary injury) is followed by a series of deleterious events that promote the progressive damage of spared tissues (secondary injury)^3^. The events involved in secondary injury include the breakdown of the blood–spinal cord barrier, haemorrhage, oedema, oxidative stress, metabolic dysfunction, glutaminergic excitotoxicity, and neuroinflammation^3–5^. The intensity of mechanical impact dictates the severity of the primary injury; however, the progression of the secondary injury may be mitigated by early pharmacological interventions following SCI. Attenuating secondary injury in SCI is a valuable therapeutic strategy because even minimal tissue sparing can have a profound impact on functional recovery^6^.

Fibrinolysis, a process in which cross-linked fibrin is degraded by plasmin, involves the conversion of plasminogen to plasmin by plasminogen activators^7^. The fibrinolytic cascade is initiated by tissue-type plasminogen activator (tPA) and/or urokinase-type plasminogen activator (uPA), and is tightly regulated by plasminogen activator inhibitor-1 (PAI-1)^8^. Over the past decades, research using mice that are deficient in these components of the fibrinolytic system have extended the known roles of this system to cover a range of functions in the central nervous system (CNS)^8–10^. A number of reports also suggest the existence of a plasmin-independent mechanism of fibrinolysis in the CNS that modulates the cellular action of astrocytes, neurons, and microglia, and which is involved in a number of pathological conditions in the CNS^7^. Although the pleiotropic functions of fibrinolysis are important for molecular biology, their diversity make it difficult to extrapolate the results obtained from the bench to the clinic. For example, *tPA*^−/−^ mice have enhanced functional recovery and reduced tissue damage after SCI^11^. In contrast, *uPA*^−/−^ mice have impaired structural remodeling of phrenic motor neuron synapses after undergoing spinal cord hemisection^12^. Therefore, the role of plasmin-dependent fibrinolysis in secondary injury in SCI and its impact on functional recovery remain unclear.

We hypothesised that the fibrinolytic system is involved in secondary injury mechanisms, because the progressive expansion of secondary haemorrhage, associated with capillary fragmentation, is one of the most destructive mechanisms of secondary injury in the CNS^5,13^. Here, we examined the role of the fibrinolytic process using a clinically relevant contusion model of SCI in mice. The objectives of this study were to: 1) reveal the effect of plasmin-dependent fibrinolytic processes on functional recovery from SCI using gene-engineered mice; and 2) develop a therapeutic strategy targeting components of the fibrinolytic system.

## Results

### Plasminogen deficiency enhances locomotor recovery in the acute phase of SCI

**Figure 1:**
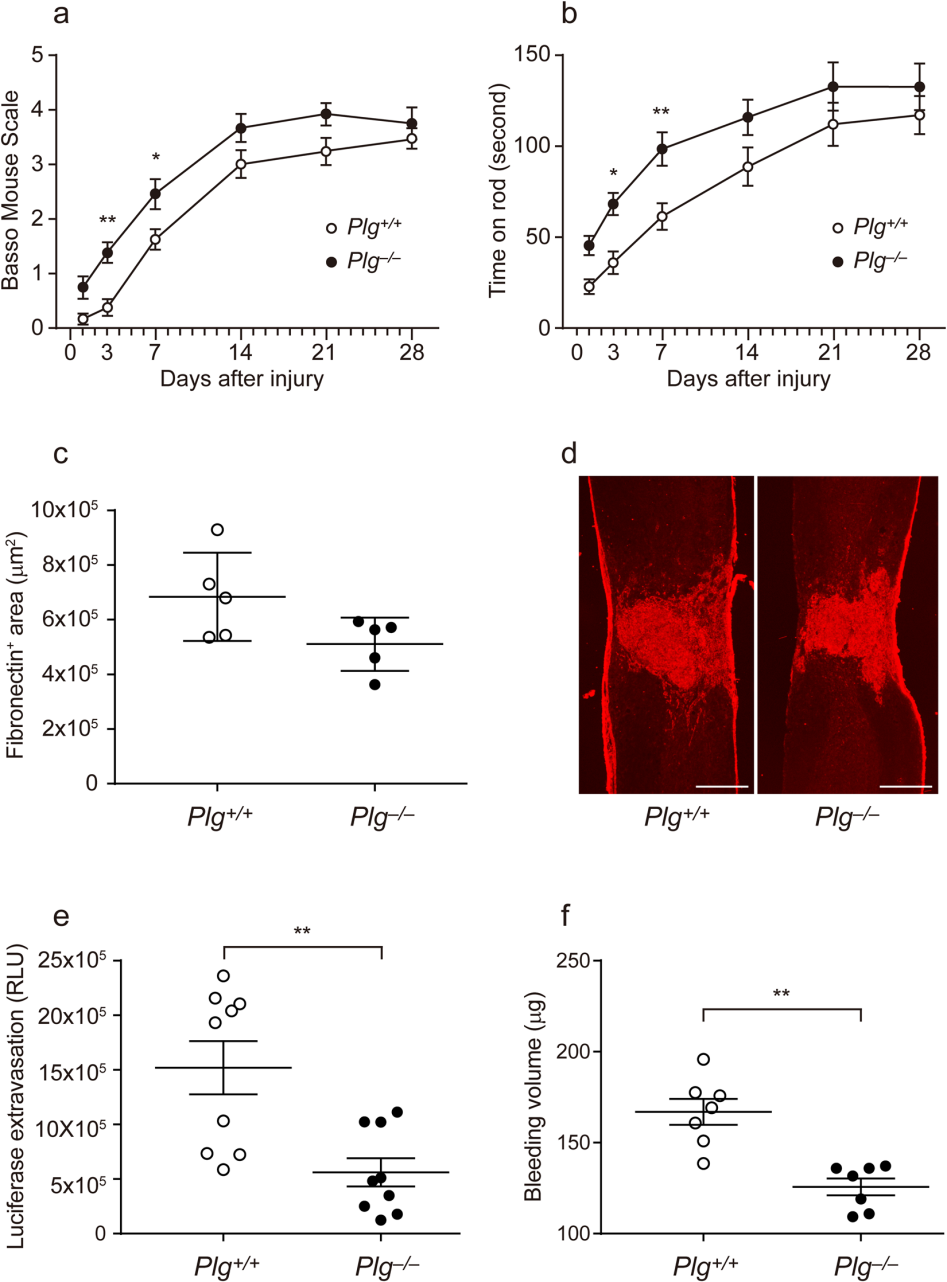
Plasminogen deficiency enhances locomotor recovery in the acute phase of SCI. Contusion SCI was induced using the Infinite Horizons impactor in *Plg*^+/+^ and *Plg*^−/−^ mice. (**a,b**) The recovery of motor function was assessed by the BMS score (**a**) and the rotarod performance test (b) at the indicated times after SCI. Values and error bars represent mean ± SEM (*n* = 12 in each group). *P < 0.05, **P < 0.01, compared between *Plg*^+/+^ and *Plg*^−/−^ mice (*post-hoc* Bonferroni test). (**c,d**) Area of fibrous scar tissue in the spinal cord was assessed by fibronectin staining at 28 dpi. (**c**) Scar area was quantified using image analysis software. Values and error bars represent mean ± SD (*n* = 5 in each group). (**d**) Representative micrographs of fibronectin staining. (**e,f**) Vascular permeability (**e**) and tissue bleeding (**f**) in the spinal cord at 1 dpi were assessed by haemoglobin concentration and luciferase extravasation, respectively. Values and error bars represent mean ± SD (*n* = 7 and 9 in each group, respectively). **P < 0.01, compared between *Plg*^+/+^ and *Plg*^–/–^ mice (Mann–Whitney *U* test in e and two-tailed Student’s *t*-test in (**f**). Scale bars: 400 μm.

We first employed plasminogen-deficient (*Plg*^−/−^) mice to investigate the role of the fibrinolytic system in locomotor recovery after SCI. We compared locomotor recovery of *Plg*^−/−^ mice with that of *Plg*^+/+^ (wild-type) mice after a contusion SCI. *Plg*^−/−^ mice had significantly better Basso Mouse Scale (BMS) scores^6^ than *Plg*^+/+^ mice at 3 and 7 days post injury (dpi; repeated-measures ANOVA followed by a *post-hoc* test); however, this statistically significant difference disappeared at 14 dpi or later (Fig. 1a). Similarly, *Plg*^−/−^ mice demonstrated significantly better locomotor performance than *Plg*^+/+^ mice in the rotarod test at 3 and 7 dpi only (Fig. 1b). Consistent with the similar locomotor function at the endpoint of our study, histological evaluation revealed that fibrous scar tissue sizes, demarcated by fibronectin staining, did not differ significantly between the groups at 28 dpi (Fig. 1c,d).

### Decreased blood–spinal cord barrier permeability and tissue bleeding in *Plg*^−/−^ mice

To determine genotype-dependent differences in early pathological changes following SCI, we assessed blood–spinal cord barrier permeability at 1 dpi using a luciferase assay. *Plg*^−/−^ mice had significantly decreased blood–spinal cord barrier permeability compared with *Plg*^+/+^ mice (Fig. 1e). We further examined the amount of tissue bleeding in the contused spinal cord at 1 dpi; *Plg*^−/−^ mice had significantly decreased tissue bleeding compared with *Plg*^+/+^ mice (Fig. 1f).

### Acute administration of tranexamic acid (TXA) promotes functional recovery after SCI

**Figure 2:**
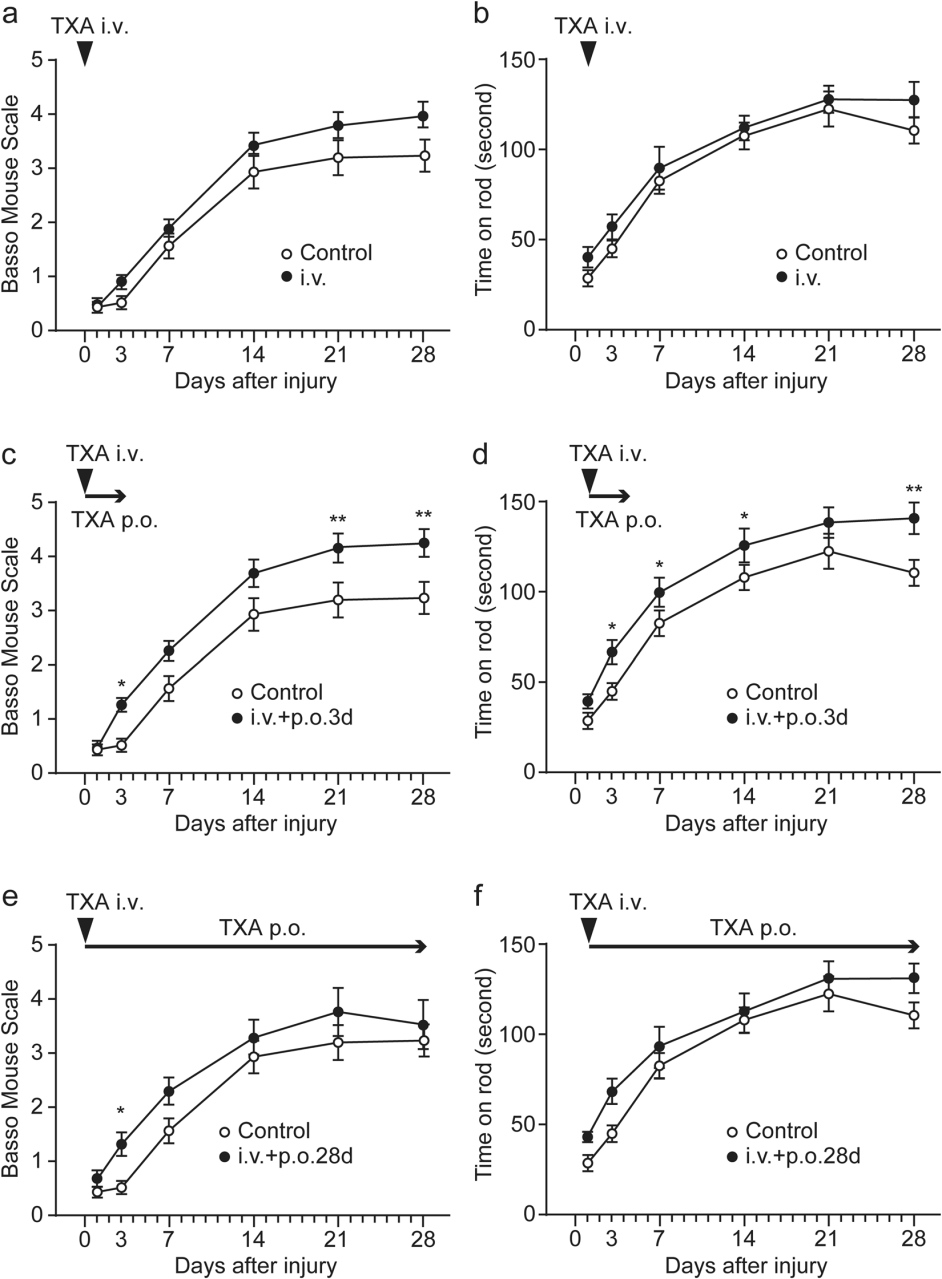
Short-term administration of tranexamic acid (TXA) promotes functional recovery after SCI. Contusion SCI was induced by the Infinite Horizons impactor in C57BL/6 mice. The mice received intravenous treatment of saline control or 100 mg/kg of TXA. Mice were then either treated without (**a,b**) or with subsequent per os administration of TXA (20 mg/mL of drinking water) for 3 days (**c,d**) or 28 days (**e,f**). The recovery of motor function was quantified by the BMS score (**a,c,e**) and the rotarod performance test (**b,d,f**) at the indicated times after SCI. Values and error bars represent mean ± SEM (*n* = 15 in each group). *P < 0.05, **P < 0.01, compared between control and TXA treatment (*post-hoc* Bonferroni test).

**Figure 3:**
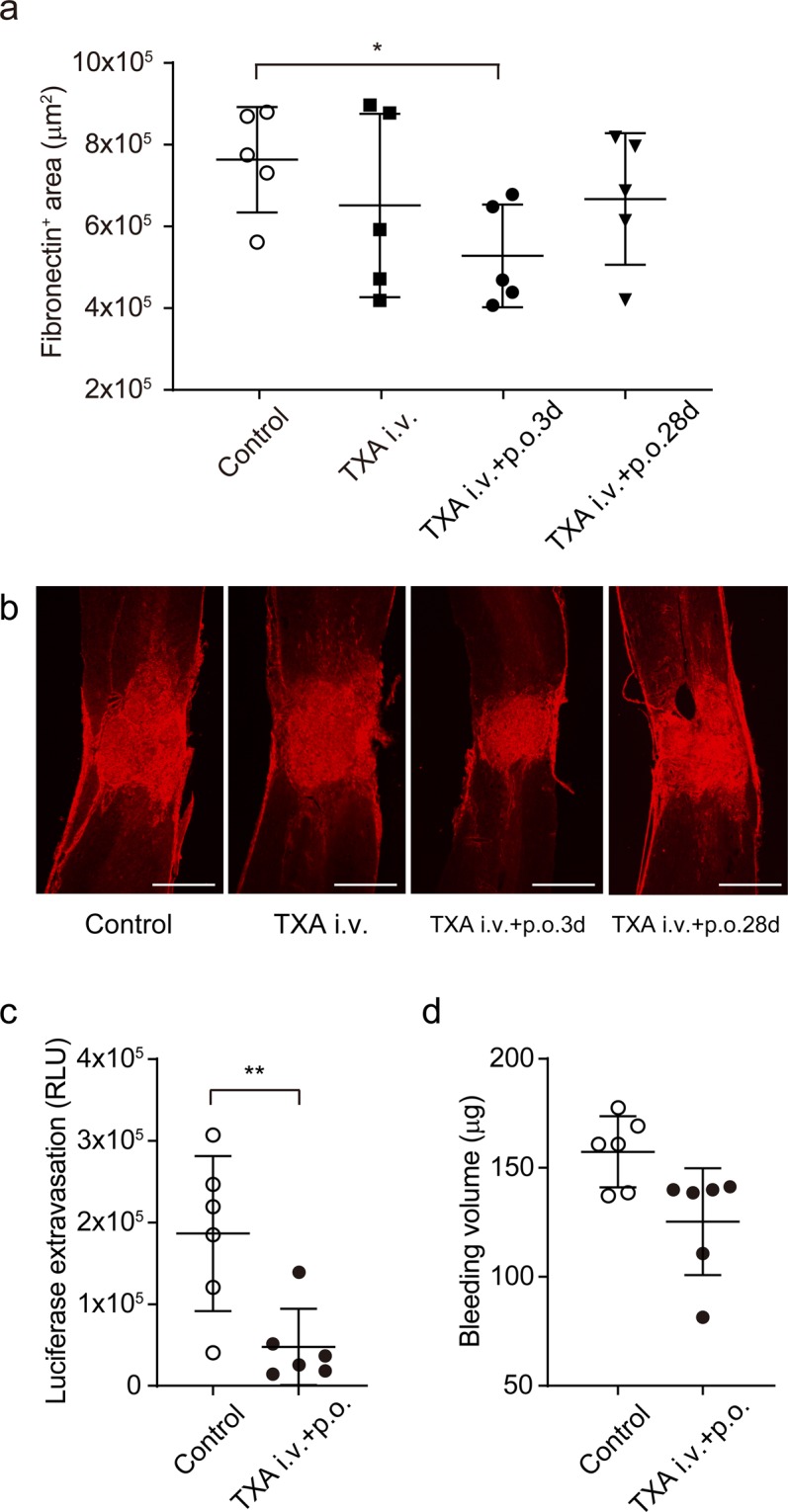
Short-term administration of tranexamic acid (TXA) reduces the area of scar tissue in the spinal cord after SCI. Contusion SCI was induced by the Infinite Horizons impactor in C57BL/6 mice. Mice were treated with a bolus intravenous injection of TXA immediately after SCI (i.v.); a bolus administration followed by per os administration of TXA for 3 days (i.v. + p.o.3d); a bolus administration followed by per os administration of TXA for 28 days (i.v. + p.o.28d); or a bolus intravenous injection of saline immediately after SCI (Control). (**a**,**b**) Area of fibrous scar tissue in the spinal cord was assessed by fibronectin staining at 28 dpi. (a) Scar area was quantified by image analysis software. Values and error bars represent mean ± SD (*n* = 5 in each group). *P < 0.05, compared between Control and i.v. + p.o.3d groups (two-tailed Student’s *t*-test). (**b**) Representative micrographs of fibronectin staining. (**c**,**d**). Vascular permeability(**c**) and tissue bleeding (**d**) in the spinal cord at 1 dpi were assessed by haemoglobin concentration and luciferase extravasation, respectively. Values and error bars represent mean ± SD (*n* = 6 in each group). *P < 0.05, **P < 0.01, compared between control and TXA treatment (Mann–Whitney *U* test). Scale bars: 400 μm.

Based on the transiently enhanced locomotor recovery in *Plg*^−/−^ mice, we hypothesised that plasminogen deficiency might exert a protective effect up to 3 to 7 dpi, but that it subsequently interferes with regeneration processes after SCI because of the important role that the fibrinolytic system plays in tissue remodelling^14^. To test this hypothesis, wild-type mice were divided into four groups according to the route and duration of TXA administration: (1) a bolus intravenous injection of TXA immediately following SCI (i.v.); (2) a bolus administration followed by per os administration of TXA for 3 days (i.v. + p.o.3d); (3) a bolus administration followed by per os administration of TXA for 28 days (i.v. + p.o.28d); and (4) a bolus intravenous injection of saline immediately following SCI (control). Mice in the i.v. + p.o.3d group showed significantly improved locomotor function assessed by both the BMS scale and the rotarod test compared with those in the control group (*P* = 0.004 and *P* = 0.005, respectively; repeated-measures ANOVA; *n* = 15 for each group; Fig. 2c,d). *Post-hoc* tests at each time point revealed that BMS scores differed significantly between these two groups at 3, 21, and 28 dpi (Fig. 2c,d). Consistent with the locomotor function results, the fibrous scar tissue areas were significantly smaller in the i.v. + p.o.3d group than those in the control group (Fig. 3a,b). We confirmed the reduced histological damage by a stereological quantitative analysis of Luxol Fast Blue (LFB) staining for myelin sparing. Mice treated with TXA (i.v. + p.o.3d) had significantly increased sparing of LFB-positive myelin compared with saline-treated control mice (*P* = 0.015; two-way repeated-measures ANOVA; *n* = 7 for each group; Supplementary Fig. 1). In contrast, continuous administration and a single intravenous injection of TXA failed to improve locomotor function (Fig. 2a,b). These data suggest that short-term inhibition of fibrinolytic activity during the acute phase of SCI improves locomotor function.

### TXA reduces blood–spinal cord barrier permeability, tissue bleeding, and production of inflammatory cytokines and chemokines

**Figure 4:**
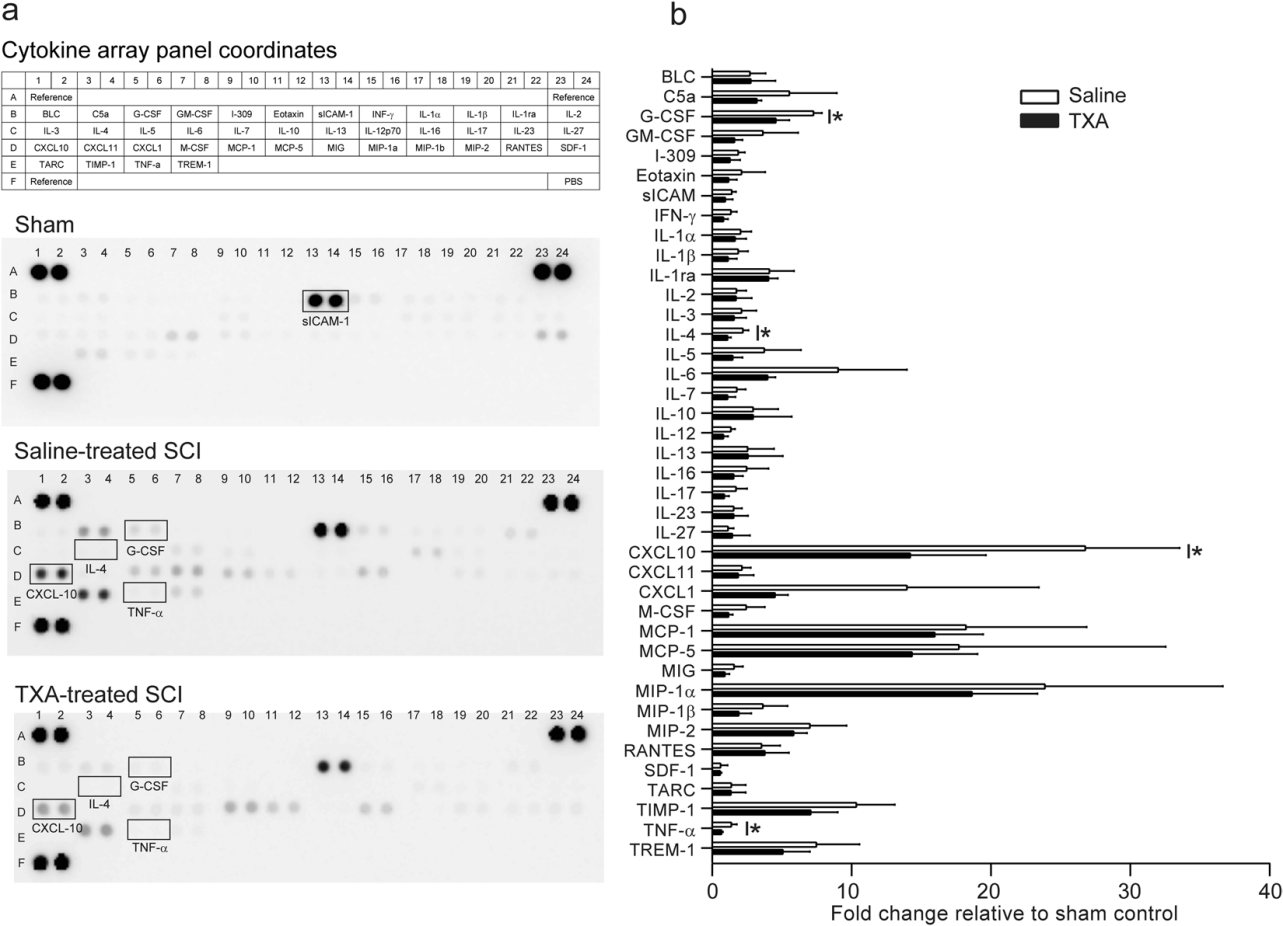
Changes in cytokine and chemokine profiles in the injured spinal cord with tranexamic acid (TXA) treatment. Contusion SCI was induced by the Infinite Horizons impactor in C57BL/6 mice treated without or with TXA. The cytokine and chemokine levels in the injured spinal cord were assessed by a Mouse Cytokine Array Panel A kit (R&D Systems) at 1 dpi. Representative blotting of cytokine array panels in sham-operated group, saline-treated SCI group, and TXA-treated SCI group (**a**). The expression of sICAM-1 in the sham control was highest among the 40 cytokines tested, but showed a small change following the induction of SCI. The intensity of each cytokine expression relative to its expression in the sham control was compared between saline-treated SCI group and TXA-treated SCI group (**c**). Values and error bars represent mean ± SD (*n* = 3 in each group). *P < 0.05, compared between saline and TXA treatment (two-tailed Student’s *t*-test).

We next examined whether TXA can improve the acute phase of secondary injury. Similar to the results obtained from *Plg*^−/−^ mice, mice treated with TXA (i.v. + p.o.3d) had significantly decreased blood–spinal cord barrier permeability compared with saline-treated mice at 1 dpi (Fig. 3c). The amount of tissue bleeding also appeared to be reduced in TXA-treated mice compared with control mice, although the difference did not reach statistical significance (*P* = 0.062, Mann–Whitney *U* test, Fig. 3d). We further measured the local concentrations of cytokines and chemokines in the injured spinal cord using protein array analysis. Forty cytokines and chemokines that are involved in the inflammatory process were detected using a Proteome Profiler Array (Fig. 4a,b). Quantification of optical density revealed that TXA administration exerted an overall suppressive effect on inflammatory cytokines and chemokines (Fig. 4b). The between-group differences were significant for TNFα, IL-4, G-CSF, and CXCL-10 (*n* = 3 for each group, *P < 0.05).

### TXA reduces activation of MMP-2 and MMP-9 in the injured spinal cord

**Figure 5:**
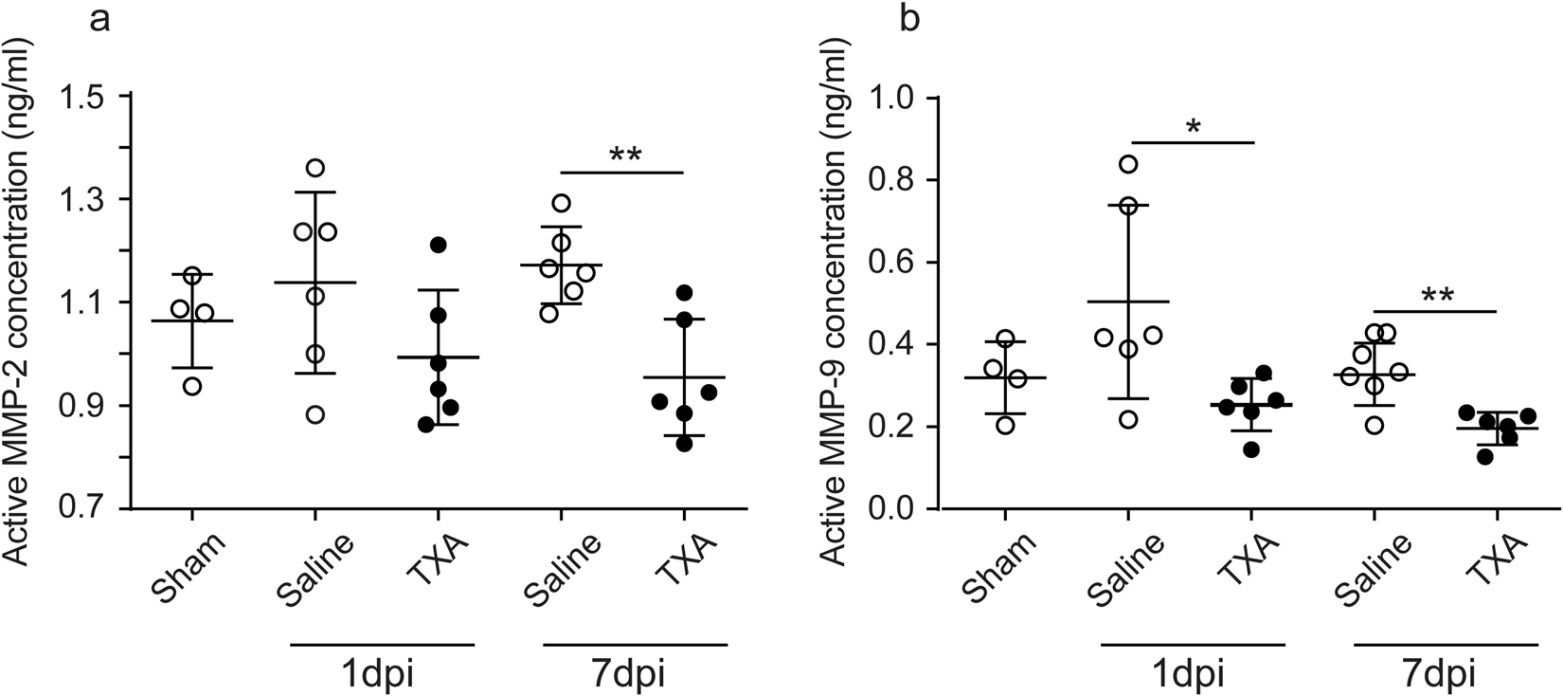
TXA reduces activation of MMP-2 and MMP-9 in the injured spinal cord. Contusion SCI was induced by the Infinite Horizons impactor in C57BL/6 mice treated without or with TXA. Mice were treated with a bolus intravenous injection of TXA just after SCI followed by per os administration of TXA (20 mg/mL of drinking water). The concentrations of active MMP-2 (**a**) and MMP-9 (**b**) in the spinal cord were assessed at day 0 (laminectomised mice without SCI; Sham), day 1 (1 dpi), or day 7 (7 dpi). Values and error bars represent mean ± SD (*n* = 6 in each group). *P < 0.05, compared between saline-treated control mice and TXA-treated mice (two-tailed Student’s *t*-test).

We lastly investigated whether TXA treatment abolished tissue remodelling after SCI. Matrix metalloproteinases (MMPs) are activated through conversion from pro-MMPs by plasmin cleavage, and are reportedly involved in tissue remodelling and regeneration after SCI^15^. We measured the levels of active MMP-2 and MMP-9 expression in the spinal cord after SCI, without or with TXA treatment (TXA i.v. + p.o.). The active forms of MMP-2 and MMP-9 at 1 dpi were lower in TXA-treated mice than in saline-treated control mice, although this difference did not reach statistical significance in the case of MMP-2 (Fig. 5a,b). The statistically significant reduction in MMP activation with TXA treatment became clearer at 7 dpi (Fig. 5a,b). These data suggest that prolonged TXA exposure inhibits MMP activation at the site of injury and thus affects resultant tissue remodelling.

## Discussion

The modulation of classical plasmin-dependent fibrinolysis offers therapeutic potential in the control of thrombosis and bleeding; recombinant tPA and TXA are widely used in clinical situations for embolic stroke and bleeding tendency, respectively^16^. To investigate plasmin-dependent roles in the pathogenesis of SCI, we used *Plg*^−/−^ mice and TXA, a lysine derivative, to inhibit the binding of plasminogen to fibrin clots. We found that *Plg*^−/−^ mice exhibited significantly improved locomotor function in the early phase of SCI, but failed to demonstrate conclusive functional recovery in the later phase. In addition, the short-term administration of TXA improved locomotor function after SCI, whereas prolonged TXA did not. Prolonged TXA administration abolished tissue remodeling probably though the inhibition of MMP activity.

The most important result in the present study is that the fibrinolytic system has a time-dependent biphasic role in the pathological process following SCI. Our detailed time-lapse examination into functional recovery after SCI allowed us to further elucidate this phenomenon. We found that in the first 7 dpi, *Plg*^−/−^ mice exhibited significantly improved locomotor function that was associated with significant reductions in bleeding and vascular permeability. Consistent with these findings, the short-term inhibition of plasmin activity by TXA treatment significantly improved locomotor function after SCI. In contrast, prolonged treatment with TXA diminished its beneficial effect, and resulted in the significant inhibition of MMP activation in the injured spinal cord. These results suggest that the fibrinolytic system exacerbates spinal cord damage in the acute phase by enhancing blood–spinal cord barrier degradation and haemorrhage; however, it also promotes tissue remodelling, probably via the activation of MMPs in the later phases of SCI.

Traumatic SCI is typically accompanied by disruption of the blood–spinal cord barrier and the subsequent expansion of the secondary haemorrhage, which is one of the most destructive mechanisms of secondary injury after SCI^5,13^. The haemorrhagic lesion extends rostrally and caudally during the hours after injury, and involves the formation of small petechial haemorrhages on the periphery of the primary lesion^17^. The haemorrhage exacerbates spinal cord damage by increasing oedema, ischaemia, and hypoxia of spared tissues^5,13,17^. Furthermore, blood itself exerts detrimental effects in the CNS through different mechanisms: First, haemoglobin, a major component of blood, induces cytotoxicity in neurons via an iron-dependent, oxidative mechanism^18^. Second, the blood protein fibrin elicits neuroinflammation in the CNS via the activation of CD11b/CD18^+^ microglia, leading to demyelination and subsequent axonal damage^19^. Therefore, reducing the amount of bleeding using anti-fibrinolytic agents, such as TXA, may protect spared neural tissue and enhance functional recovery after traumatic SCI.

SCI elicits a robust intraspinal inflammatory cascade arising from the activation of innate immune cells and infiltrating leukocytes^3^. The inflammatory process plays a pivotal role in the development of secondary damage, and controlling neuroinflammation is an important therapeutic approach for SCI^20^. Neuroinflammatory processes, however, appear to be a dual-edged sword: they can either be deleterious, through exacerbating neuronal damage, or beneficial, through enhancing regenerative events^21^. In the present study, TXA significantly reduced the expression of several cytokines and chemokines in the spinal cord, including TNF-α, IL-4, G-CSF, and CXCL-10, which suggests that the inhibition of fibrinolysis by TXA modulates neuroinflammation after SCI. The reduced expression of TNF-α and CXCL-10 is theoretically beneficial for regeneration because these pro-inflammatory cytokines exacerbate neuroinflammation and interfere with functional recovery after SCI^20^. However, the reduced expression of IL-4 and G-CSF may interfere with neurological recovery because these cytokines are neuroprotective^22,23^. Further study is thus required to conclude whether TXA exerts a beneficial or detrimental influence on the modulation of neuroinflammation after SCI.

The weakened functional recovery observed in *Plg*^−/−^ mice and in wild-type mice treated with continuous TXA administration may be partly attributable to the suppression of local proteolytic activity that is required for tissue remodelling and neuronal regeneration in the later phases of SCI. The fibrinolytic system enhances extracellular matrix remodelling and cell migration through the activation of MMPs^7^. Therefore, the prolonged administration of TXA beyond 7 dpi may impair the CNS remodelling process, which is mediated by the activation of MMPs. In agreement with our hypothesis, Noble *et al*.^15^ demonstrated that the short-term administration of GM6001, a general inhibitor of MMPs, promoted functional recovery after SCI; however, this beneficial response was lost when the treatment was extended to the first week. Fibrinolysis-dependent degradation of the extracellular matrix is also thought to promote axonal regrowth and synaptic plasticity after SCI^24,25^. In addition, fibrinolysis facilitates the clearance of fibrin deposition, which induces neuroinflammation and subsequent demyelination via microglial activation. Taken together, the inhibition of fibrinolysis in the later phases of SCI may interfere with neurological recovery.

Our data, obtained from mouse experiments, indicate the possibility of TXA as an acute treatment strategy for SCI. TXA has received considerable attention for its ability to control severe bleeding in various clinical situations. TXA successfully reduced mortality and transfusion in severe trauma patients with bleeding (in the CRASH-2 trial)^26^, and the CRASH-3 trial is currently in progress to quantify the effects of early TXA administration on death and disability in patients with traumatic brain injury^27^. It has also been shown that TXA reduces death caused by bleeding in women with post-partum haemorrhage (WOMAN trial)^28^. In both the CRASH-2 trial and the WOMAN trial, the administration of TXA was less effective with increasing delay, and was even detrimental for survival if it was not administered within 1–3 h^26,28^. Thus, TXA should be administrated as soon as possible after an event onset^28^. This is consistent with our finding that inhibition of the fibrinolytic system at a later phase of SCI hinders functional recovery.

In conclusion, the fibrinolytic system has time-dependent biphasic actions following SCI. Our results indicate that the temporally optimised modulation of fibrinolytic activity may be a novel therapeutic strategy to improve functional outcome after traumatic SCI. Because there are currently limited treatment options to restore function in the damaged spinal cord, it is very important to develop novel therapeutic approaches. TXA has been widely used as an antifibrinolytic drug in clinical situations for many years, and the long-term safety of the drug has been well established. The abundant experience in its use will make it easy to apply the drug in a clinical study of SCI. However, further experiments using larger animals and clinical trials are required to optimise the timing of initiation, dosage, and duration of TXA administration for its application in SCI treatment.

## Methods

### Animals

Plasminogen-deficient (*Plg*^−/−^) mice were provided by Dr. P. Cameliet (University of Leuven, Leuven, Belgium)^29^, bred on a C57BL/6J background (B6.129S2-*Plg*^+/−^), and maintained by heterozygous breeding. For experiments involving drug administration, C57BL/6J female mice were purchased from Japan SLC (Shizuoka, Japan). All animal procedures were approved by the Institutional Animal Care and Concern Committee of Jichi Medical University (Tochigi, Japan), and animal care was performed in accordance with the guidelines of the committee.

### Mouse model of contusion spinal cord injury

Animals were anaesthetised using isoflurane and a laminectomy was performed at the T10 vertebral level. Contusion SCI was induced using the Infinite Horizons impactor (Infinite Horizons, L.L.C., Lexington, KY, USA) using a force of 60 kdyn. Postoperative care was performed as previously described^30^. Sham-operated mice received a T10 level laminectomy without spinal cord contusion.

### Locomotor function

The recovery of open-field locomotor performance was evaluated using the nine-point BMS, which is widely used to evaluate hindlimb motor function in mice^31^. Mice in an open field were observed individually for 4 min each by two investigators blinded to the group status (Y.S. and A.K.), and hindlimb motor function was scored according to BMS guidelines. Recovery of motor function was also quantified using a rotarod performance test (MK-610A; Muromachi Kikai Co., Tokyo, Japan). The rotarod treadmill consists of a computer-controlled stepper motor-driven drum (diameter 30 mm) with either constant or accelerating speed modes; when the animal falls, the amount of time spent by the animal on the drum is automatically recorded. We measured ride performance in the acceleration speed mode (30 rpm/300 s) to assess motor function after SCI at the indicated times. Two trials were performed by each mouse, and analysis was performed using the average results. Both the BMS and the rotarod test were assessed at 1, 3, 7, 21, and 28 dpi. We confirmed that laminectomy does not affect locomotor functions. Sham-operated mice (*n* = 3) had a score of 9 (full points) in the BMS at all time points. In the rotarod test, there was no significant difference between naïve and sham-operated mice at 1 day post operation (301.3 ± 30.4 vs. 295.0 ± 47.7, *P* = 0.856).

### Drug administration

For pharmacological inhibition of the fibrinolytic system we used TXA, a lysine analogue that reversibly inhibits plasminogen binding via kringle domains^32^. A bolus injection of saline containing TXA (100 mg/kg; Merck KGaA, Darmstadt, Germany) or saline alone was administered immediately after the contusion injury was induced. For continuous dosing, mice received per os administration of TXA via drinking water at a concentration of 20 mg/mL.

### Tissue processing

At 28 dpi, mice that were deeply anaesthetised using isoflurane were perfused with 50 mL of phosphate-buffered saline (PBS) followed by 50 mL of 4% paraformaldehyde. The isolated spinal cord was fixed with 4% paraformaldehyde in PBS for 2 h at 4 °C, and then immersed in 30% sucrose for 48 h. Spinal cords were cut into 5 mm segments centred on the injury site before being frozen in the presence of optimal cutting temperature compound (Sakura Finetek, Torrance, CA, USA). Serial longitudinal sections (20 μm thick) or cross sections (10 μm thick) of the cord were prepared through the lesion site from frozen tissues at −25 °C and mounted on poly-L-lysine-coated glass slides (Matsunami, Osaka, Japan).

### Measurement of lesion size

For measurement of the lesion scar size, five to seven serial sections (20 μm thick) containing the lesion epicentre were analysed per animal, as described previously^33,34^. Fibronectin staining was performed to delineate the lesion border of fibrous scarring^35^. Sections were permeabilised with 0.3% Triton X-100, blocked with 5% normal horse serum in 0.1 M phosphate buffer, and incubated with primary antibody to fibronectin (1:200; Merck KGaA) at 4 °C overnight. After rinsing with PBS, sections were incubated for 1 h at room temperature with a species-specific secondary antibody conjugated with Alexa 594 (ThermoFisher Scientific, Waltham, MA, USA), and then mounted in VECTASHIELD Mounting Medium with DAPI (Vector Laboratories, Burlingame, CA, USA). Images were obtained using a fluorescence microscope (BZ-9000; Keyence, Osaka, Japan). Measurement of the fibronectin-positive area was performed at the lesion epicentre using an image analysis software (VH-H1A5; Keyence, Osaka, Japan). For quantification of spared white matter, Luxol fast blue (LFB) staining was performed on serial cross sections (10 μm thick) and areas of spared white matter were calculated using the Cavalieri method. Briefly, a point grid was made and superimposed onto images of the cross sections at 200 µm intervals, centred at the lesion epicentre. The epicentre was defined as the section containing the least amount of spared white matter. Points included in the spared white matter were counted using image analysis software (VH-H1A5) and point tallies were converted into area estimates using the following formula:

$${\rm{area}}={\rm{a}}/{\rm{p}}\times \sum {\rm{P}},$$

where a/p equals the area represented by each point, and ∑P equals the number of points counted in each image. The percentage of spared white matter area was calculated by dividing the measured areas of spared myelin by the average white matter area at the T10 level of sham-operated controls.

### Blood–spinal cord barrier permeability

To quantify blood–spinal cord barrier permeability of proteins, mice were anaesthetised with isoflurane, and 80 µl of a 0.5 µg/µL solution of luciferase (Merck KGaA) in PBS containing 0.001% bovine serum albumin was injected into the jugular vein 30 min before processing^36^. Immediately after flushing out blood by perfusing with 100 mL of PBS, a 5 mm block of the spinal cord (epicentre ± 2.5 mm) was collected. The luciferase activity of the spinal cord tissue was measured using a luciferase assay kit (Promega, Madison, WI, USA) as described previously^37^. The intensity of luminescence was expressed as a relative light unit (RLU).

### Tissue bleeding

Mice were sacrificed at 1 dpi and perfused with heparinised saline to remove intravascular blood. A freshly dissected spinal cord (epicentre ± 2.5 mm) was homogenised in distilled water (250 μL) and processed to measure tissue bleeding, as previously described^38^. Briefly, 20 μL of supernatant containing haemoglobin was incubated with 80 μL of Drabkin’s reagent (Merck KGaA), and the haemoglobin concentration was assessed by measuring the optical density of the solution at a 550 nm wavelength.

### MMP-2 and MMP-9 activity assays

At each time point, mice were deeply anaesthetised with isoflurane, and a 5 mm length of spinal cord centred at the injury epicentre was immediately dissected for MMP activity assay. Dissected spinal cords were homogenised in Tris-HCl buffer containing 0.1% Triton-X-100, and assessed using mouse MMP-2 and MMP-9 activity assays (QuickZyme Biosciences, Leiden, the Netherlands). Samples and standards were added to a microplate that was pre-coated with MMP-2 or MMP-9 capture antibody. The detection enzyme and substrate were added to each well. Absorbance at 405 nm was read at 0, 6, and 22 h using a Spark 10 M multimode plate reader (TECAN, Männedorf, Switzerland). A standard curve was used to determine the concentration of MMP-2 or MMP-9 in each sample (ng/mL).

### Cytokine and chemokine array

Tissue sections of the spinal cord (5 mm) were harvested in 500 µL of PBS containing cOmplete^®^ protease inhibitor cocktail, sonicated, and then lysed by the addition of Triton X-100 (final concentration 1%). After the freeze–thaw cycle, samples were centrifuged at 10,000 × *g* for 5 min to remove cell debris. The relative changes of spinal cord cytokine and chemokine concentrations following SCI were measured by the Proteome Profiler Mouse Cytokine Array Panel A Kit (R&D Systems, Minneapolis, MN, USA), according to the manufacturer’s recommendation. To exclude the effect of surgical procedure, we employed a sham-operated group (laminectomised mice without SCI; n = 3) as an internal control. The intensity of each cytokine and chemokine expression in the injured spinal cord was expressed as a fold change to its expression in the sham-operated group.

### Statistical analysis

Unless otherwise stated, values are expressed as the mean ± SD. Assumptions of parametric statistical tests, such as normal data distribution (Shapiro–Wilk test) and homoscedasticity (Levene’s test), were assessed in cases in which Student’s *t*-tests or repeated-measures analysis of variance (ANOVA) were used. All datasets passed the normality tests except (1) luciferase extravasation (Figs 1e and 3c), and (2) bleeding volume (Fig. 3d). The nonparametric Mann–Whitney *U* test was employed to compare these data. Differences were considered significant at *P* < 0.05. All data were analysed using statistical software (IBM SPSS Statistics Version 25.0, IBM corp., Armonk, NY, USA).

## Supplementary information

Supplementary Figure 1
