# Supplementary material for: Short-term inhibition of fibrinolytic system restores locomotor function after spinal cord injury in mice
Source: Sci Rep. 2019 Nov 5;9:16024. doi: 10.1038/s41598-019-52621-8 (PMC6831600; doi:10.1038/s41598-019-52621-8)
Supplement: Supplementary file 1 — Supplementary Figure 1 [file 41598_2019_52621_MOESM1_ESM.pdf]

Short-term inhibition of fibrinolytic system restores locomotor function after spinal cord injury in mice

Yasuyuki Shiraishi[1,*], Atsushi Kimura[1,*,**], Osamu Matsuo[2], Yoichi Sakata[3], Katsushi Takeshita[1], Tsukasa Ohmori[3]

[1]Department of Orthopaedics, Jichi Medical University School of Medicine, Tochigi, 329-0498, Japan.

[2]Kindai University Faculty of Medicine, Osakasayama, 589-8511, Japan.

[3]Department of Biochemistry, Jichi Medical University School of Medicine, Tochigi, 329-0498, Japan.

[*]These two authors contributed equally to this work.

**Corresponding Author: Atsushi Kimura, M.D., Ph.D. (akimura@jichi.ac.jp)

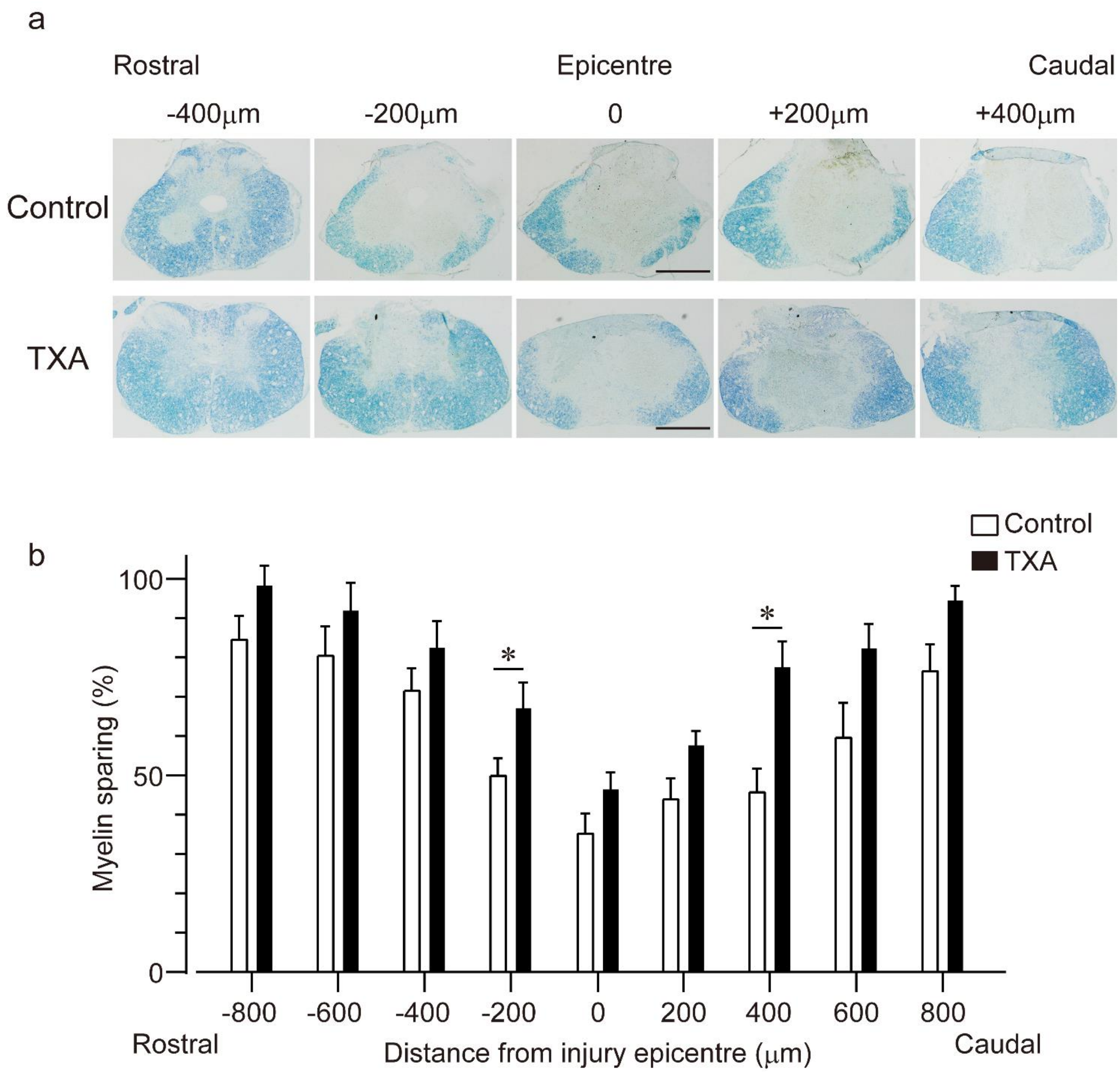

Supplementary Figure 1. Stereological quantification of spared myelin. The extent of Luxol fast blue (LFB)-positive myelin sparing was compared between saline-treated control and TXA-treated (i.v. + p.o.3d) mice at 28 dpi. (a) Representative micrographs of LFB staining in control and TXA-treated mice at various distances rostral and caudal to the injury epicentre. (b) LFB-positive myelin-spared areas were compared between control and TXA-treated mice. Values and error bars represent mean ± SEM (*n* = 7 in

each group). *$P < 0.05$, (two-way repeated measures ANOVA with *post-hoc* Bonferroni test). Scale bars: 400 μm.
